# Suicide risk among veterans using VHA justice-involved services: a latent class analysis

**DOI:** 10.1186/s12888-023-04725-9

**Published:** 2023-04-07

**Authors:** Ryan Holliday, Adam R. Kinney, Alexandra A. Smith, Jeri E. Forster, Matthew A. Stimmel, Sean C. Clark, Shawn Liu, Lindsey L. Monteith, Lisa A. Brenner

**Affiliations:** 1 Department of Veterans Affairs, Rocky Mountain Regional Medical Center, Rocky Mountain Mental Illness Research, Education and Clinical Center for Suicide Prevention, 1700 N. Wheeling St, 80045 Aurora, CO USA; 2 University of Colorado Anschutz Medical Campus, Colorado, USA; 3 Veterans Health Administration Homeless Programs Office, National Center on Homelessness among Veterans, Washington, DC USA; 4 Veterans Health Administration Homeless Programs Office, Veterans Justice Programs Office, Washington, DC USA; 5 Veterans Health Administration Homeless Programs Office, Washington, DC USA

**Keywords:** Justice involvement, Veteran, Suicide, Latent class analysis

## Abstract

**Background:**

Justice-involved Veterans experience notable risk for psychosocial stressors (e.g., homelessness) and psychiatric multimorbidity, which can result in complex clinical presentations. However, research examining how such factors coalesce to impact risk for suicide remains limited.

**Methods:**

We conducted a latent class analysis of 180,454 Veterans accessing Veterans Health Administration (VHA) justice-related services from 2005 to 2018.

**Results:**

A four-model class membership solution was identified. Among these classes, risk for suicide was highest among Veterans with greater psychiatric burden, with risk most notable among those with high VA service use. Veterans seeking healthcare primarily focused on substance use disorders or with low psychiatric burden and service use had a lower risk for suicide.

**Conclusions:**

Psychiatric multimorbidity is salient as it relates to suicide among Veterans accessing VHA justice-related services. Further evaluation of existing VHA services for this population and methods of augmenting and enhancing care for justice-involved Veterans with histories of co-occurring psychiatric conditions may be beneficial in facilitating suicide prevention efforts.

**Supplementary Information:**

The online version contains supplementary material available at 10.1186/s12888-023-04725-9.

## Background

According to the most recent Bureau of Justice Statistics report, Veterans comprise approximately 8% of individuals currently in prison within the United States [1]. Moreover, a sizable proportion of Veterans are currently on parole or probation, suggesting the percentage of Veterans with any interaction with the criminal justice system is likely much higher [2].

While rates of criminal justice system involvement among Veterans do not appear to be elevated relative to non-Veteran adults, justice-involved Veterans differ from justice-involved non-Veterans in several ways. In particular, justice-involved Veterans have higher documented rates of violent offenses compared to justice-involved non-Veteran adults [1, 3–5]. For example, in a recent report by Department of Justice [1], they noted that male Veterans in state (69.3%) and federal (24.7%) prisons had significantly higher rates of violent crime offenses relative to male non-Veterans (state: 56.7%; federal: 12.6%). Such offenses result in lengthier sentencing, societal stigma, and difficulty transitioning back into the community following incarceration [6–8].

Justice-involved Veterans also often experience psychiatric conditions and symptoms (e.g., substance use, serious mental illness, posttraumatic stress disorder). Data suggest that justice-involved Veterans have higher rates of several conditions and symptoms than both non-Veterans involved with the justice system and Veterans without a history of criminal justice involvement [9–13]. In addition, justice-involved Veterans experience psychosocial stressors (e.g., housing and occupational difficulties) with high propensity, which can further impede functioning [14]. Decreased psychosocial functioning (e.g., unemployment), coupled with stigma related to legal involvement, can impact perceptions of oneself, the world, and the future, which may subsequently influence risk for suicide [6, 15].

To meet the needs of justice-involved Veterans, the Veterans Health Administration (VHA) has developed a number of programs specifically tailored for this population [16]. For example, Veterans Justice Outreach (VJO) and Health Care for Reentry Veterans programs focus on connecting and engaging justice-involved Veterans in health and social services. Although these programs often prove beneficial to recovery, the Veteran population that accesses VJO and Health Care for Reentry Veterans services often have a high concentration of suicide risk factors [17]. Potential factors among Veterans accessing these services that may contribute to elevated suicide risk include psychiatric multimorbidity and stressors (e.g., housing instability). Such risk factors may result in clinical complexity and difficulties engaging Veterans in healthcare, thus impacting ability to participate and benefit from intervention. In fact, when psychiatric conditions and housing instability were accounted for in a prior examination of suicide risk among Veterans accessing VHA justice-related services, the effect of justice involvement substantially attenuated and, at times, was no longer significant [17].

Given the clinical complexity and presence of multiple drivers of suicide risk among members of this population, multivariate analytic approaches capable of identifying groups at risk is warranted. Specifically, latent class analytic approaches have demonstrated the ability to identify phenotypes of individuals based on a number of factors (e.g., psychiatric diagnosis, trauma exposure, service use). As such, our team combined multiple national datasets to identify Veterans accessing VHA justice-related services who did and did not die by suicide. A latent class analytic approach was used to understand how health comorbidity, psychiatric conditions, and VHA service use coalesced to generate latent classes among the VHA-using justice-involved Veteran population. Such classes were then examined as they related to suicide mortality to inform which Veterans were at greatest risk for suicide.

## Methods

### Data source and participants

The current study was conducted using data collected via a retrospective chart review of Veterans’ VHA electronic health records (EHR). We identified justice-involved Veterans based on any use of VHA justice-related services (i.e., Veterans Justice Outreach; Healthcare for Re-entry Veterans)^1^ between 1/2005 and 12/2018.^2^

For this cohort, we obtained data regarding sociodemographics (i.e., age, race, ethnicity, rurality), service connection, psychiatric diagnoses (i.e., posttraumatic stress disorder [PTSD], adjustment disorder, anxiety disorder, depressive disorder, bipolar disorder, alcohol use disorder, substance use disorder, personality disorder; schizophrenia, and other psychotic disorder), documented history of traumatic brain injury (TBI), military sexual trauma (MST; from the universal VA MST screen), and medical comorbidity (i.e., Charlson Comorbidity Index [18]). Diagnoses were identified using ICD codes (VHA EHR).^3^ In addition, we accessed data regarding use of VHA mental health care, overall VHA care, and homeless services.^4^ Mental health care, as well as VHA use (not including mental health or justice-related services), were identified using stop and bed codes and categorized based on tertile values for the distribution of frequency of unique visits over the study period: *mental health care* - low (≤ 30, moderate (> 30 and ≤ 141), or high (> 141); and *overall use* - low (≤ 65), moderate (> 65 and ≤ 185), or high (> 185) use. Due to high levels of homeless service engagement among justice-involved Veterans, VHA homeless service use was also included [14], which we examined as a dichotomous variable (yes/no).

VHA EHR data were combined with data from the U.S. Veterans Eligibility Trends and Statistics (USVETS) and VA/DoD Mortality Data Repository (MDR) National Death Index data. Sociodemographic data from USVETS was cross-referenced with VHA EHR data to enhance the validity of the dataset. MDR was used to determine mortality status within our cohort (who was alive versus had died, including by suicide or other causes).

In total, we identified 197,737 justice-involved Veterans with available data for the current examination. Given the focus on suicide mortality, we removed 17,283 Veterans classified by MDR as having died by a non-suicide-related cause. This resulted in a final cohort for the latent class analysis of 180,454 Veterans, 822 (0.5%) of whom died by suicide from 2005 to 2018. The current study was approved by the Colorado Multiple Institutional Review Board.

### Analytic Plan

#### Class enumeration

Class enumeration was performed using Mplus v.8.5 [19]. We used latent class analysis to identify subgroups of justice-involved Veterans based on psychiatric diagnosis, documented TBI, MST screening results, medical comorbidity, and VHA service use (i.e., overall, mental health, and homeless service use). Latent class analysis is a mixture modelling approach which uses categorical observed variables to identify unobserved, mutually exclusive subgroups within a sample [20]. Response patterns for observed variables are used to classify individuals into mutually exclusive latent classes [21]. We fit models with one through eight latent classes, relying on information criteria (e.g., AIC; BIC), the Lo-Mendell-Rubin (LMR) adjusted likelihood ratio test, and clinical interpretability to select a parsimonious, statistically sound, and theoretically plausible model [20, 22]. Conditional item probabilities were used to characterize and label the classes. Such probabilities represent the likelihood of experiencing the observed trait (e.g., PTSD) assuming membership in the unobserved (i.e., latent) class. Upon selecting the model with the optimal class solution, we conducted a descriptive analysis of covariates among the total sample and stratified by class membership. A Veteran’s class membership was considered the class for which the Veteran had the highest likelihood of membership relative to other classes (i.e., posterior membership probabilities).

#### Latent class membership as a predictor of suicide

We then examined the indicator of Veterans’ class membership, identified using posterior membership probabilities, as a predictor of death by suicide. Unadjusted logistic regression was used to model suicide (yes/no) as a function of latent class membership to determine if the risk for suicide differs across classes (note that given the rare outcome, the odds ratio is a reasonable estimate of the relative risk and we therefore interpret results as such). Subsequently, we fit an adjusted logistic regression model, where suicide was modeled as a function of latent class membership, age (i.e., 18–39 years, 40–49 years, 50–59 years, 60–69 years, ≥ 70 years), sex, race (i.e., White, Black, non-Black underrepresented racial group [comprised of American Indian/Alaska Native, Asian, Native Hawaiian/Pacific islander, and “Other” given relatively small sample sizes for each]), ethnicity, rurality, and service connection (yes/no). Statistical significance of all parameter estimates was evaluated at *α* = 0.05. Additionally, due to the size of the sample, both 95% and 99% CI are reported.

## Results

Table 1 includes sample characteristics of the cohort. Overall, the cohort was largely male (93.9%), White (64.4%), and non-Hispanic (91.4%). Psychiatric diagnoses were highly prevalent within our sample, with substance use disorder (65.2%), alcohol use disorder (63.1%), and depressive disorders (63.6%) the most frequent. In addition, documented medical comorbidity was low for most of the sample (76.6%). More than half of justice-involved Veterans in our sample had a history of VHA homeless service use (62.0%).

**Table 1:** Sample Characteristics of Veterans Using VHA Justice-Related Services (N = 180,454)

Variable	*n*	%
Age		
18–39	36,867	20.4
40–49	30,646	17.0
50–59	48,524	26.9
60–69	48,598	26.9
≥70	15,819	8.8
Sex		
Male	169,006	93.9
Female	10,919	6.1
Race		
White	116,260	64.4
Black	55,309	30.6
American Indian/Alaska Native	2,501	1.4
Asian	1,235	0.7
Native Hawaiian/Pacific islander	205	0.1
Other	4,944	2.7
Ethnicity		
Hispanic	14,458	8.1
Non-Hispanic	164,998	91.9
Rural	24,830	13.9
Service connected	19,202	10.6
PTSD	89,450	49.6
Adjustment disorder	72,808	40.3
Anxiety disorder	92,887	51.5
Depressive disorder	114,722	63.6
Bipolar disorder	31,835	17.6
Alcohol use disorder	113,920	63.1
Substance use disorder	117,686	65.2
Personality disorder	35,809	19.8
Schizophrenia	19,897	11.0
Other psychotic disorder	25,662	14.2
Positive MST screen	15,751	8.7
Documented TBI	29,203	16.2
Charlson Comorbidity Index		
0	138,140	76.6
1	26,264	14.6
≥2	16,050	8.9
VHA homeless service use	111,843	62.0

*Note.* Data were missing as follows: sex (*n* = 529), ethnicity (*n* = 998), and rural (*n* = 1,507). Charlson Comorbidity Index calculated for study time frame. MST = military sexual trauma; PTSD = posttraumatic stress disorder; TBI = traumatic brain injury; VHA = Veterans Health Administration

### Class enumeration

The LMR adjusted likelihood ratio test was not statistically significant for the three-class solution (*p* = 0.333), indicating that adding a third class did not improve model fit to a statistically significant degree compared to the two-class solution. This provided statistical evidence in favor of the two-class solution. However, examination of information criteria (e.g., Bayesian Information Criterion) indicated that the addition of classes improved model fit, with incremental improvement beginning to diminish upon adding a fourth class (see Supplementary Table 1). Thus, we considered classes three through five as candidate class solutions. Upon carefully inspecting the conditional item probabilities for classes three through five, we chose the four-class solution because it was the most parsimonious model and included classes that were both qualitatively distinct and clinically relevant. In addition, conditional probabilities for the four-class solution were characterized by superior class separation and within-class homogeneity relative to the three- and five-class solutions. Further, average probability for most likely class membership was high across classes, ranging from 0.76 to 0.93. See Table 2 for conditional item probabilities for the four-class solution.

**Table 2:** Conditional Item Probabilities Across Latent Classes for the Four-Class Solution

	Latent Classes (%)			
Variable	High Psychiatric Burden/High Service Users (30.27%)	High Psychiatric Burden/Moderate Service Users (29.79%)	Low Psychiatric Burden/Low Service Users (25.30%)	Primarily Substance Use Related (14.64%)
**Psychiatric conditions**				
PTSD	**0.777**	**0.704**	0.113	0.191
Adjustment disorder	**0.608**	0.439	0.161	0.335
Anxiety	**0.804**	**0.688**	0.125	0.270
Depression	**0.945**	**0.824**	0.148	0.480
Bipolar disorder	0.398	0.124	0.012	0.113
Alcohol use disorder	**0.916**	**0.612**	0.228	**0.763**
Substance use disorder	**0.949**	**0.613**	0.221	**0.843**
Personality disorder	0.477	0.128	0.009	0.100
Schizophrenia	0.250	0.030	0.012	0.147
Other psychotic disorder	0.327	0.066	0.009	0.141
**Military sexual trauma**	0.177	0.092	0.018	0.019
**Medical conditions**				
TBI	0.278	0.222	0.028	0.045
Comorbidity: 0	**0.699**	**0.803**	**0.846**	**0.696**
Comorbidity: 1	0.192	0.125	0.089	0.185
Comorbidity: ≥ 2	0.109	0.072	0.065	0.119
**Service use**				
MH use: low	0.000	0.253	**0.957**	0.126
MH use: moderate	0.111	**0.688**	0.043	**0.566**
MH use: high	**0.889**	0.059	0.000	0.308
VHA use: low	0.003	0.329	**0.825**	0.192
VHA use: moderate	0.225	**0.508**	0.139	**0.525**
VHA use: high	**0.772**	0.163	0.036	0.283
Homeless service use	**0.895**	**0.502**	0.289	**0.834**

The *High psychiatric burden*^5^*/high service user* (30.27%) group was characterized by a high probability of PTSD, adjustment disorder, depression, anxiety, alcohol use disorder, and other substance use disorder. Veterans in this class were likely to receive high levels of mental health (i.e., > 141 visits) and overall VHA (i.e., > 185 visits) services and were highly likely to receive VHA homeless services. This class was distinguished from the *High psychiatric burden/moderate service user* group, which was also prevalent (29.79%), and carried a similar psychiatric burden but were most likely to receive moderate levels of mental health services (i.e., > 30 and ≤ 141 visits) and VHA (i.e., > 65 and ≤ 185 visits) services. Additionally, this class had a moderate probability (0.502) of receiving VHA homeless services. The *Low psychiatric burden/low service user* (25.53%) group was characterized by a low likelihood of all psychiatric conditions, and members were likely to receive low levels of mental health (i.e., ≤ 30 visits) and VHA (i.e., ≤ 65 visits) services. Members of this class were unlikely to receive homeless services. Finally, the *Primarily substance use disorder* (13.3%) group had a high probability of alcohol use disorder and substance use disorder, but not other psychiatric conditions, and were most likely to receive moderate levels of mental health and VHA services. Additionally, they were highly likely to receive homeless services.

#### Latent class membership as predictor of suicide

A crude logistic regression model was conducted to determine the relationship between latent class membership and suicide (see Table 3). The *Low psychiatric burden/low service user* group was chosen as the reference given prior research noting the association between psychiatric diagnosis and suicide risk (see Supplementary Table 2 for comparisons with other latent classes as the reference group).

**Table 3:** Crude and Adjusted Models Examining Class Membership as it Relates to Suicide Risk

	Crude model			Adjusted model		
Variable	OR	95%CI	99%CI	OR	95%CI	99%CI
Class membership						
Low psychiatric burden/low service users	--	--	--	--	--	--
Primarily substance use-related	0.77*	0.60, < 1.00	0.55, 1.08	0.99	0.58, 1.72	0.48, 2.04
High psychiatric burden/moderate service users	1.29**	1.07, 1.55	1.01, 1.65	1.47*	1.01, 2.14	0.89, 2.41
High psychiatric burden/high service users	1.22*	1.01, 1.47	0.95, 1.56	1.88***	1.30, 2.73	1.15, 3.07

* *p* < 0.05 ** *p* < 0.01 *** *p* < 0.001

*Note.* Models adjusted for age, sex, race, ethnicity, rurality, and service connection.

Overall, the crude model was significant (χ^2^(3) = 22.82, *p* < 0.001, Nagelkerke R^2^ = 0.002). The groups experiencing the greatest risk for suicide were the *High psychiatric burden/moderate service user* (1.29 times greater risk for suicide relative to the *Low psychiatric burden/low service user* group, *p* = 0.0057) and the *High psychiatric burden/high service user* groups (1.22 times greater risk for suicide relative to the *Low psychiatric burden/low service user* group, *p* = 0.038). Interestingly, in the crude model, Veterans in the *Primarily substance use-related* group had lower (i.e., 0.77 times) risk for suicide relative to Veterans with low psychiatric burden and service use (*p* = 0.049).

The model was subsequently adjusted for demographic factors (i.e., age, sex, race, ethnicity, rurality) and service connection (Table 3). Again, the overall model was significant (χ^2^(12) = 128.95, *p* < 0.001, Nagelkerke R^2^ = 0.036). Notably, with the inclusion of these factors, risk appeared to increase largely within the high psychiatric burden and high service use group. Specifically, Veterans in the *High psychiatric burden/high service user* group had 1.88 times greater risk for suicide compared to the *Low psychiatric burden/low service user* group (*p* = 0.001). Similarly, Veterans in the *High psychiatric burden/moderate service user* group had 1.47 times greater risk for suicide relative to the *Low psychiatric burden/low service user* group (*p* = 0.046). However, differing from the crude model, there was no significant difference in risk for suicide between the *Primarily substance use-related* and *Low psychiatric burden/low service user* groups (*p* > 0.05).

## Discussion

Building upon prior work noting risk for suicide among justice-involved Veterans [15, 17], this study provides the first latent class analysis of factors associated with risk for suicide among Veterans using VHA justice-involved services. Four latent classes were identified, with risk most pronounced in adjusted models among justice-involved Veterans experiencing high psychiatric burden and moderate to high VHA service use.

Such risk is consistent with the notion that psychiatric clinical complexity may indeed be a primary factor in driving suicide risk among justice-involved Veterans. Given the psychiatric multimorbidity and stressors that justice-involved Veterans experience, these Veterans likely present to time-limited care settings with myriad psychosocial concerns. As such, it may be difficult for VHA providers working with these Veterans to balance provision of services, including suicide prevention care [14]. For example, intervention may focus on a current stressor (e.g., upcoming eviction) rather than providing an evidence-based mental health treatment (e.g., cognitive behavioral therapy for depression). While addressing the most pressing stressor is often a critical first component of treatment, especially if it is the Veteran’s preference, additional assessment of psychiatric need could help address underlying diagnoses and inform future treatment planning [14]. Given this, identifying optimal service delivery that can reduce patterns of psychosocial stress (e.g., housing or unemployment) as well as psychiatric symptoms among this population of Veterans is needed.

Despite this, the relationship between service delivery and suicide risk in this cohort of Veterans was complex. Specifically, no significant differences in risk for suicide were found between moderate and high service groups of Veterans experiencing high psychiatric burden (see Supplementary Table 2). A potential explanation for this is that type of VHA service, rather than frequency of use, may be more relevant to understanding suicide risk. Indeed, the VHA services used in this sample could have varied substantially in potential to address and prevent suicide risk (e.g., evidence-based psychotherapy for a psychiatric diagnosis). Moreover, we generated a total number of appointments accessed in the study time frame, but this number was broad and not indicative of treatment adherence nor engagement. Therefore, an initial step to understanding the utility of existing programing with respect to suicide risk is likely a mixed-method investigation of extant VHA services accessed by justice-involved Veterans. In particular, further understanding of specific VHA services which can reduce subsequent risk for suicide, as well as Veteran reported barriers and facilitators to engaging in those services, is likely necessary to ensure that these Veterans receive optimal suicide prevention efforts.

Interestingly, risk did not substantially differ between justice-involved Veterans in the *Low psychiatric burden/low service use* and *Primarily substance use-related* groups. Several potential explanations exist for this. First and foremost, it is possible that existing VHA substance use programing may be beneficial for this subset of Veterans in decreasing risk. Indeed, Ilgen and colleagues [23] noted that Veterans who completed substance use treatment had decreased subsequent risk for suicide. In addition, it is also possible that other psychiatric diagnoses germane to the high psychiatric burden groups (e.g., depression, PTSD) may have been a larger driver of suicide risk than substance use-related diagnoses. Nonetheless, additional research remains necessary to elucidate drivers of risk within each of our identified groups.

VHA homeless service use was associated with group membership in both the high psychiatric burden and primarily substance use-related groups. Such an association makes sense given prior research documenting the relationship between mental health conditions and first-time as well as chronic homelessness [24]. However, given differing suicide risk between these groups, additional research is likely needed to determine how housing instability may differentially impact those with more psychiatric burden.

Finally, medical comorbidity (Charlson Comorbidity Index [18]), TBI, and MST did not emerge as factors distinguishing latent classes. This finding is surprising given that these factors are associated with psychiatric conditions, service use, and suicide [25–28]. Such results may in part be related to medical and other comorbidities not being identified among members of this cohort. Additional research is warranted and may benefit from efforts to examine severity of TBI (e.g., mild, moderate, severe) and type of MST (e.g., sexual harassment or sexual assault), as well as whether these factors differentially impact justice-involved Veterans’ suicide risk based on gender (e.g., association of MST in women versus men).

Despite the strength of findings, results should be interpreted within the context of limitations. First, this examination was specific to Veterans accessing VHA justice-related services. Because of this, findings may not generalize to Veterans who do not qualify for comprehensive VHA care or who interact with the criminal justice system and who do not access VHA justice-related services. Moreover, a sizable portion of our sample was moderately to highly engaged in VHA services, including mental health care. As such, many of these Veterans may have been routinely accessing services which focused on addressing psychiatric conditions as well as which assessed the presence of suicide risk factors. In a similar vein, Veterans accessing mental health services would be more likely to have a psychiatric diagnosis conferred on their EHR.

We relied on the VHA EHR, rather than conducting diagnostic interviews. As such, we are unable to determine the accuracy of diagnoses. In addition, we examined substance use disorder diagnoses as a unidimensional construct. This approach limits results given differing risk for suicide based on type of substance (e.g., opioid vs. cocaine [29]), as well as factors which may have moderated suicide risk (e.g., early vs. sustained remission).

An additional consideration is that we were unable to employ contemporary approaches to predicting distal outcomes while accounting for measurement error in latent class membership. Specifically, while we attempted to use the 3-step BCH method to examine the relation between latent class membership and death by suicide [30], we were unable to achieve model convergence, likely due to the low base rate of suicide mortality. Given this, we instead examined the relationship between class membership and suicide mortality using binary logistic regression, which may have minimized understanding of the contribution of important covariates (i.e., age, sex, race, ethnicity, rurality, service connection). It’s worth noting that the latent classes associated with the interpreted model exhibited good measurement properties, including acceptable average posterior probabilities among those assigned to each class (0.76-0.93) which exceeded the acceptable range of 0.70 [20], limiting concerns regarding measurement error biasing the results. Nonetheless, future research, inclusive of a larger suicide mortality sample, should consider examining the effect of latent class membership upon suicide among justice-involved Veterans while utilizing approaches capable of accounting for latent class measurement error.

Finally, we used EHR codes to identify use of VHA justice-related and homeless services, rather than using Homelessness Operations and Management and Evaluation System (HOMES) data [31]. Given that HOMES is an administrative database that tracks use of VA homeless services separate from the EHR, some justice-involved Veterans may not have been captured in our cohort.

Justice-involved Veterans experience several risk factors for suicide (e.g., psychiatric diagnoses, homelessness). We provided the first latent class analysis of suicide mortality among members of this population. We found that psychiatric burden and moderate to high service use are factors associated with risk. Investigation of current and novel programing for this population may be a potential method of enhancing suicide prevention efforts for this at-risk population of Veterans.

## Electronic supplementary material

Below is the link to the electronic supplementary material.

Supplementary Material 1 Supplementary Table 1: Model fit indices for models with ≤ 8-class solutions

Supplementary Material 2 Supplementary Table 2: Crude and Adjusted Models with Other Latent Classes as Reference Group

## Data Availability

All data are available upon request contingent upon IRB and VA guidelines and approval.

## Footnotes

1 Justice-involved services were determined based on outpatient stop codes (591, 592) mirroring the method from Palframan and colleagues [17].

2 Timeframe was determined based on data completeness (U.S Veterans Eligibility Trends and Statistics complete from 2005 onward) and availability (National Death Index data available through 2018) at the time of analysis.

3 Health conditions were confirmed based on presence of 1 inpatient or 2 outpatient documented encounters.

4 Homeless services were determined based on ICD codes (ICD-9: V60.0; ICD-10: Z59.0), outpatient stop codes (501, 504, 507, 508, 511, 522, 528, 529, 530, 590), and inpatient specialty codes (28, 29, 37, and 39).

5 “Psychiatric burden” is indicative of risk for psychiatric diagnoses (i.e., posttraumatic stress disorder [PTSD], adjustment disorder, anxiety disorder, depressive disorder, bipolar disorder, alcohol use disorder, substance use disorder, personality disorder; schizophrenia, and other psychotic disorder).
