# Supplementary material for: Suicide risk among veterans using VHA justice-involved services: a latent class analysis
Source: BMC Psychiatry. 2023 Apr 7;23:235. doi: 10.1186/s12888-023-04725-9 (PMC10080851; doi:10.1186/s12888-023-04725-9)
Supplement: Supplementary file 1 — Supplementary Material 1 Supplementary Table 1: Model fit indices for models with ≤ 8-class solutions [file 12888_2023_4725_MOESM1_ESM.docx]

| Supplementary Table 1.  *Model fit indices for models with ≤ 8-class solutions* | | | | | | |
| --- | --- | --- | --- | --- | --- | --- |
| **Class Solution** | **LL** | **AIC** | **BIC** | **ABIC** | **LMR p-value** | **Entropy** |
| 1 | -1811962.677 | 3623963.353 | 3624155.315 | 3624094.932 | – | – |
| 2 | -1605581.520 | 3211241.040 | 3211635.066 | 3211511.123 | < .001 | 0.854 |
| 3 | -1559740.665 | 3119599.329 | 3120195.420 | 3120007.915 | 0.333 | 0.817 |
| 4 | -1548378.879 | 3096915.757 | 3097713.913 | 3097462.847 | < .001 | 0.765 |
| 5 | -1538841.567 | 3077881.135 | 3078881.355 | 3078566.728 | < .001 | 0.758 |
| 6 | -1531338.857 | 3062915.714 | 3064117.998 | 3063739.811 | < .001 | 0.741 |
| 7 | -1525155.236 | 3050588.472 | 3051992.821 | 3051551.073 | < .001 | 0.749 |
| 8 | -1519560.306 | 3039438.613 | 3041045.027 | 3040539.718 | < .001 | 0.739 |
