# Supplementary material for: Suicide risk among veterans using VHA justice-involved services: a latent class analysis
Source: BMC Psychiatry. 2023 Apr 7;23:235. doi: 10.1186/s12888-023-04725-9 (PMC10080851; doi:10.1186/s12888-023-04725-9)
Supplement: Supplementary file 2 — Supplementary Material 2 Supplementary Table 2: Crude and Adjusted Models with Other Latent Classes as Reference Group [file 12888_2023_4725_MOESM2_ESM.docx]

Supplementary Table 2

*Crude and Adjusted Models with Other Latent Classes as Reference Group.*

|  | Odds of Suicide Relative to Reference Class (OR/95%CI/99%CI) | | | |
| --- | --- | --- | --- | --- |
|  | Reference Class (Crude) | | | |
|  | 1 | 2 | 3 | 4 |
| 1. Low psychiatric burden/low service users | - | 1.30*/>1.00,1.68/.92,1.83 | .78**/.64,.93/.61,.99 | .82*/.68,.99/.64,1.05 |
| 2. Primarily substance use-related | - | - | .60***/.47,.76/.43,.83 | .63***/.49,.81/.46,.87 |
| 3. High psychiatric burden/moderate service users | - | 1.67***/1.31,2.14/1.21,2.31 | - | 1.06/.90,1.25/.85,1.32 |
| 4. High psychiatric burden/high service users | - | 1.58***/1.24,2.02/1.15,2.19 | .95/.80,1.12/.76,1.18 | - |
|  | Reference Class (Adjusted) | | | |
| 1. Low psychiatric burden/low service users | - | 1.01/.58, 1.74/.49,2.07 | .68*/.47,.99/.41,1.12 | .53***/.37,.77/.33,.87 |
| 2. Primarily substance use-related | - | - | .68/.41,1.13/.35,1.32 | .53*/.32,.87/.27,1.02 |
| 3. High psychiatric burden/moderate service users | - | 1.48/.89,2.46/.76,2.89 | - | .78/.58,1.05/.53,1.16 |
| 4. High psychiatric burden/high service users | - | 1.89*/1.15,3.12/.98,3.65 | 1.28/.95,1.73/.87,1.90 | - |

* *p <* 0.05 ** *p* < 0.01 *** *p <* 0.001

*Note.*  Models adjusted for age, sex, race, ethnicity, rurality, and service connection.
